# Repertoire, Genealogy and Genomic Organization of Cruzipain and Homologous Genes in *Trypanosoma cruzi*, *T. cruzi*-Like and Other Trypanosome Species

**DOI:** 10.1371/journal.pone.0038385

**Published:** 2012-06-07

**Authors:** Luciana Lima, Paola A. Ortiz, Flávia Maia da Silva, João Marcelo P. Alves, Myrna G. Serrano, Alane P. Cortez, Silvia C. Alfieri, Gregory A. Buck, Marta M. G. Teixeira

**Affiliations:** 1 Departamento de Parasitologia, ICB, Universidade de São Paulo, São Paulo, São Paulo, Brasil; 2 Department of Microbiology and Immunology, Medical College of Virginia Campus of Virginia Commonwealth University, Richmond, Virginia, United States of America; 3 Faculdade de Farmácia, Universidade Federal de Goiás, Goiânia, Goiás, Brasil; Federal University of São Paulo, Brazil

## Abstract

*Trypanosoma cruzi,* the agent of Chagas disease, is a complex of genetically diverse isolates highly phylogenetically related to *T. cruzi*-like species, *Trypanosoma cruzi marinkellei* and *Trypanosoma dionisii,* all sharing morphology of blood and culture forms and development within cells. However, they differ in hosts, vectors and pathogenicity: *T. cruzi* is a human pathogen infective to virtually all mammals whilst the other two species are non-pathogenic and bat restricted. Previous studies suggest that variations in expression levels and genetic diversity of cruzipain, the major isoform of cathepsin L-like (CATL) enzymes of *T. cruzi*, correlate with levels of cellular invasion, differentiation, virulence and pathogenicity of distinct strains. In this study, we compared 80 sequences of genes encoding cruzipain from 25 *T. cruzi* isolates representative of all discrete typing units (DTUs TcI-TcVI) and the new genotype Tcbat and 10 sequences of homologous genes from other species. The catalytic domain repertoires diverged according to DTUs and trypanosome species. Relatively homogeneous sequences are found within and among isolates of the same DTU except TcV and TcVI, which displayed sequences unique or identical to those of TcII and TcIII, supporting their origin from the hybridization between these two DTUs. In network genealogies, sequences from *T. cruzi* clustered tightly together and closer to *T. c. marinkellei* than to *T. dionisii* and largely differed from homologues of *T. rangeli* and *T. b. brucei*. Here, analysis of isolates representative of the overall biological and genetic diversity of *T. cruzi* and closest *T. cruzi*-like species evidenced DTU- and species-specific polymorphisms corroborating phylogenetic relationships inferred with other genes. Comparison of both phylogenetically close and distant trypanosomes is valuable to understand host-parasite interactions, virulence and pathogenicity. Our findings corroborate cruzipain as valuable target for drugs, vaccine, diagnostic and genotyping approaches.

## Introduction

Cathepsin L-like (CATL) are cysteine proteases that play important roles in cell invasion, growth, differentiation, immunity, immune-modulation, virulence, pathogenicity and survival of pathogenic protozoans. Different isoforms of CATL are encoded by a large gene family and perform distinct roles in the interactions of the trypanosomes with vertebrate hosts and vectors, differing in stage, cellular localization and expression level during the life cycle. This functional and structural diversification may have contributed to the adaptation of different trypanosome species to their different life cycles, vertebrate hosts and vectors [1]–[4].

*Trypanosoma cruzi* is the type species of the subgenus *Schizotrypanum* and a complex of genetically heterogeneous isolates distributed in 6 intraspecific subdivisions denominated discrete typing units (DTUs), TcI-TcVI [5], [6], and one new genotype (Tcbat) identified in Brazilian bats [7]. Closest relatives of *T. cruzi* are the bat-restricted *T. c. marinkellei* followed by *T. dionisii,* which are referred as *T. cruzi*-like due to morphologically indistinguishable blood and culture forms [8]–[11]. Development within mammalian cells *in vitro* is a feature shared by all species of the subgenus *Schizotrypanum*, while *in vivo* only *T. cruzi* has been proven to infect mammals other than chiropterans [7], [9], [10], [12]–[14]. Bats infected by *T. cruzi*-like species show nests of amastigotes in cardiac, skeletal and stomach muscle cells likewise *T. cruzi* in a range of hosts including man. Recent studies demonstrated that *T. dionisii* and *T. cruzi* invade mammalian cells through a common mechanism involving lysosome mobilization to the site of parasite entry [15], [16]. Previous studies showed that *T. cruzi* and *T. dionisii* share similar molecules with important roles in host-parasite interactions such as phospholipids and cysteine proteases [17], [18] as well as epitopes associated to autoimmunity in Chagas disease [19]. Nevertheless, *T. dionisii* differ from *T. cruzi* in surface glycoproteins involved in host-cell interactions [16]. Besides morphology and *in vitro* and *in vivo* behavior, *T. cruzi*-like species share cellular, biochemical and immunological features with *T. cruzi* and, hence, can be valuable as non-infective to humans models for studies of *T. cruzi* and as targets for trials of drugs, vaccines and diagnosis [10], [20].

Similar to *T. cruzi*, all *T. cruzi*-like isolates differentiate from epimastigotes to infective metacyclic trypomastigotes in cultures and in the vector gut; bats are infected by licking vector feces contaminated with trypanosomes on their fur and/or by ingesting the infected vectors themselves [8], [9]. Differently from *T. cruzi* that can be transmitted by several genera of triatomine bugs, transmission of *T. c. marinkellei* seems to involve only triatomines of the genus *Cavernicola,* while cimicids are vectors of *T. dionisii* [8], [9]. In addition to *T. cruzi*, *T. rangeli* was reported infecting humans and non-human primates, chiropterans and mammals of other orders and are both transmitted by triatomines [21], [22]. The mechanisms underpinning vertebrate and vector specificities of these trypanosomes are unknown.

Previous studies demonstrated that two main CATL enzymes are expressed by *T. cruzi*, the major isoform (>75 copies) addressed in the present study and designated as cruzipain, is the archetype of a large multigene family organized in tandem repeats expressed in all life cycle stages of *T.* cruzi [4], [23]–[26]. Analysis of polymorphic cruzipain-encoding genes disclosed the isoform cruzipain 2 (∼6 copies), which is expressed preferentially by the mammalian stages and differs markedly from cruzipain with respect to substrate specificity and kinetic properties [27], [28].

Cruzipain plays fundamental functions in *T. cruzi* life cycle with recognized roles in parasite-host interactions, in establishing, maintaining, exacerbating and controlling infections. There are increasing evidence that the immunopathogenesis of experimental Chagas Disease is, at least in part, due to the activity of cruzipain mediating cell invasion, inflammation, tissue damage and immune evasion [29], [30]. Cruzipain is an immunodominat antigen, expressed on parasite surface and secreted, which elicits potent humoral [31], [32] and cellular immune responses in *T. cruzi* infected humans and mice [33], [34]. Vaccination with recombinant cruzipain trigger strong humoral and cell-mediated immunity controlilng parasite load and inflammatory tissue damage [34]–[37]. Addition of synthetic irreversible inhibitors to cultures of cells infected with *T. cruzi* blocks parasite replication, intracellular growth and differentiation [38], [39]. Treatment of *T. cruzi* infected mice with inhibitors designed to inactivate cruzipain rescued mice from a lethal infection [40]. *T. cruzi* ability to invade human cells was modulated by the balance between cruzipain and chagasin, a natural endogenous inhibitor of papain-like cysteine proteases [41], [42]. Therefore, inhibitors of cruzipain are among the most promising new drugs for treatment of Chagas disease [1], [4], [43]–[45].

Studies have suggested that the variable levels of cruzipain activity correlate to degrees of metacyclogenesis, cellular invasion and virulence of *T. cruzi* isolates. Comparison of TcI and TcII strains suggested that cruzipain proteolytic profiles could be useful for separating members of these two DTUs and that high expression levels could be linked to enhanced metacyclogenesis and cell infectivity [46]. In a study using flow cytometry and anti-cruzipain antibody both TcI and TcII isolates showed heterogeneous surface cruzipain patterns, however, expression levels were higher in TcI isolates showing higher metacyclogenesis [47]. In *T. cruzi* Dm28c (TcI), cruzipain are down regulated during metacyclogenesis [48]. The over expression of this enzyme throughout the parasite life-cycle was associated with enhanced metacyclogenesis but not with increased cell infectivity [49]. Differences in cruzipain expression were correlated with differential virulence for mice of *T. cruzi* isolates of Z3 (TcIII and IV) [50]. A proteomic analysis suggested that significant differences in the expression of cruzipain by isolates of TcIII and TcIV (Z3) could also contribute to their differential infectivity to cells [51]. Interestingly, proteolytic activity was lower in the virulent *T. cruzi* CL strain compared to the non-virulent CL-14 clone [52]. Overall, these studies pointed toward noteworthy but still controversial association between levels of cruzipain expression/activity and virulence, metacyclogenesis and cell infectivity of *T. cruzi* strains. Low levels of activity and transcripts of CATL-like in *T. rangeli* (rangelipain) were related to lack of pathogenicity and intracellular development [53]. However, a broad study demonstrated that isolates of divergent lineages of *T. rangeli* express high levels of enzymatic activity and transcripts of rangelipain [54].

The major cruzipain isoform has been the subject of extensive biochemical, structural and immunological studies. However, to date, sequences of cruzipain-encoding genes were not yet comparatively examined in *T. cruzi* isolates of all DTUs displaying different degrees of cellular invasion, virulence and pathogenicity. Despite limited to a few strains and lacking sequence analysis, studies suggested relevant diversity of cruzipain genes [55], [56]. Genetic diversity and activity of enzymes from *T. cruzi* of all DTUs and *T. cruzi*-like species need to be investigated. An understanding of the expression, repertoires and evolutionary relationships of genes encoding cruzipain in *T. cruzi* of different DTUs and closest related *T. cruzi-*like species, and comparison with *T. rangeli* and *T. b. brucei,* which are phylogenetically distant from *T. cruzi* with life cycles (which are phylogenetically distant from *T. cruzi* and unable to develop within mammalian cells in vitro), whose life cycles differ in vertebrate hosts and vectors and differing in vertebrate hosts and vectors, can assist in clarifying the potential role of these enzymes in the host-parasite interactions, virulence and pathogenicity.

Comparative studies between closely related pathogenic (*T. cruzi*) and non-pathogenic trypanosomes (*T. cruzi*-like) can contribute for understanding the evolution of the pathogenicity and virulence of *T. cruzi* and can be helpful for successful design of control, diagnostic and genotyping strategies. With these purpose, our goals in this study were: to characterize the repertoire of genes encoding cruzipain from isolates of all *T. cruzi* DTUs and compare them with homologues from *T. c. marinkellei, T. dionisii, T. rangeli* and *T. b. brucei*; to analyze the relationships among these genes by network genealogies; to investigate their genomic organization by synteny analysis of loci containing cruzipain genes in trypanosome genomes; to assess the expression of cruzipain homologues by the non-pathogenic *T. cruzi*-like species by northern-blotting and proteolytic assays.

## Results

### Comparison of whole Genes Encoding CATL Enzymes in Distinct *T. cruzi* DTUs, *T. dionisii, T. rangeli* and *T. b. brucei*

Like mammalian papain-like enzymes, CATL enzymes of trypanosomatids are synthesized as inactive precursors, consisting of pre, pro, and catalytic domains (cd), and a C-terminal extension. Proteolytic cleavage of the N-terminal pro-domain generates the mature enzyme consisting of a cd domain and a C-terminal extension unique of trypanosomatids [24], [26]. To compare the entire cruzipain sequences (∼450 amino acids), sequences from *T. cruzi* Sylvio X10.6 and G (TcI), Y and Esmeraldo cl3 (TcII), M6241 cl6 (TcIII), CL Brener (TcVI, one sequence from each the Esmeraldo-like and non-Esmeraldo-like haplotypes) and from Tcbat 1994 were aligned with homologous sequences from *T. c. marinkellei* 344 and *T. dionisii* 211. Sequences from non-*Schizotrypanum* species (*T. rangeli* and *T. b. brucei*) were included in the alignments [54].

**Figure 1:**
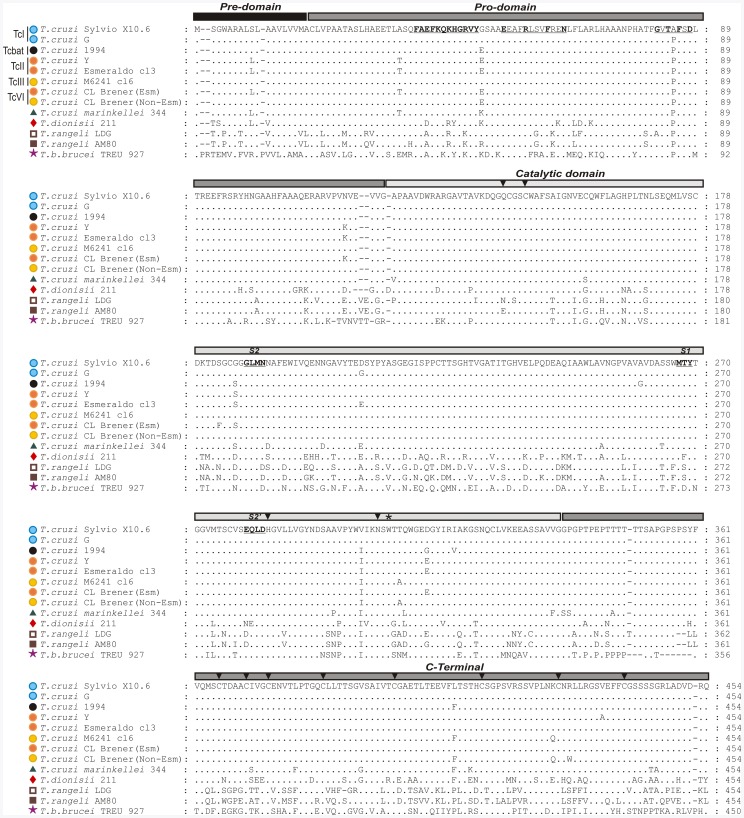
Alignment of predicted amino acid sequences from entire cruzipain of *T. cruzi* (TcI, TcII, TcIII, TcVI and Tcbat) and homologues from *T. cruzi*-like (*T. c. marinkellei* and *T. dionisii*), *T. rangeli* and *T. b. brucei*. Pre, pro, catalytic domain and C-terminal extension amino acid sequences of cruzipain genes from *T. cruzi* Sylvio X10.6 and G (TcI), TCC1994 (Tcbat), Y and Esmeraldo cl3 (TcII), M6241 cl6 (TcIII), CL Brener (TcVI) Non-Esmeraldo-like (TcIII) and Esmeraldo-like (TcII) haplotypes and homologues from *T. c. marinkellei* (344), *T. dionisii* (211), *T. rangeli* (LDG and AM80) and *T. b. brucei* (TREU 927). The CATL family signatures of pro-domain motifs ERFININ (ERFN) and GNFD (GTFD) are indicated in bold and underlined, the subsites S1, S2 and S2′ are in bold, and the conserved Trp181 are indicated by (*).The glutamine [Q] of the oxyanion hole, cysteine [C], histidine [H] and asparagine [N] of catalytic triad in the catalytic domain, and 8 cysteines in the C-terminal extension are indicated by arrow heads.

Overall identities were high in the N-terminal region, either in pre- and pro-domains (∼94 and 91%, respectively) and catalytic domains (∼90%), and most variable in the C-terminal regions (∼85%) (Fig. 1). As typically found in peptidases of Clan CA, which are targeted to intracellular compartments and secreted, all cruzipain and homologous genes have a signal peptide at their N-terminal region, as well as the catalytic triad of cysteine, histidine and asparagine residues (Cys25, His159 and Asn179) and the highly conserved Trp181. Important sites for autocatalytic cleavage, the motifs ERFNIN-like and GNFD-like of pro-domains are conserved in all trypanosomes. The clan CA is characterized by having substrate specificity defined by the S2 pocket. In cruzipain genes from all *T. cruzi* DTUs and homologues from *T. c. marinkellei*, the S2 subsites are conserved whereas in *T. dionisii, T. rangeli* and *T. b. brucei* divergent amino acids were found in these regions, suggesting differences in substrate specificities (Fig. 1).

**Figure 2:**
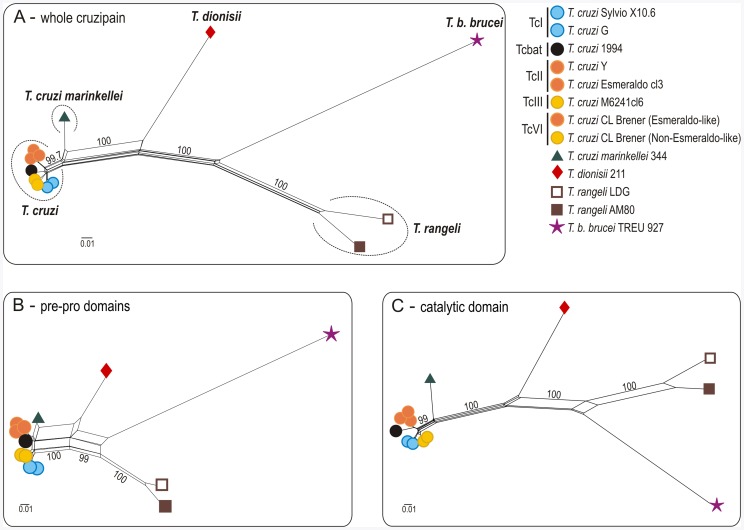
Network genealogies of predicted amino acid sequences from all domains of genes encoding cruzipain in *T. cruzi* and homologues in other trypanosome species. Networks produced using the Neighbour-Net algorithm in SplitsTree v4.11.3, excluding all conserved sites and with Uncorrected p-distance. Networks were produced using entire sequences (A), pre- and pro-domains (B) or restricted to catalytic domains (C) of cruzipain encoding genes from the different trypanosomes are indicated by different symbols and colors according to the legend. Numbers in nodes correspond to support values estimated by performing 100 bootstrap replicates using the same parameter optimized for network inferences.

Cruzipain amino acid sequences from *T. cruzi* isolates were relatively conserved in all domains. The ratio of non-synonymous (*dN*) to synonymous (*dS*) substitutions in the catalytic domain was *dN/dS* <1 by comparing the distinct DTUs of *T. cruzi* and *T. cruzi*-like species, suggesting that the enzymatic domain of cruzipain genes has been subjected to stabilizing selection for the conservation of metabolic function within the subgenus *Schizotrypanum*. Sequences encoding homologous cruzipain genes of *T. c. marinkellei* were closely related to those of *T. cruzi* (∼6.5% divergence) but the divergences were larger than those separating the *T. cruzi* DTUs (maximum of ∼2.5%). Sequences from *T. cruzi* largely diverged from homologues of *T. dionisii* (∼20%), *T. rangeli* (∼33%) and *T. b. brucei* (∼43%) in all domains (Fig.1). Genealogies based on whole cruzipain genes, or restricted to pre-pro or to catalytic domains, resulted in identical topologies (Fig. 2).

### Genealogy of Genes Encoding *T. cruzi* Cruzipain and Homologues from *T. c. marinkellei*, *T. dionisii* and Non-*Schizotrypanum* Trypanosome Species

**Figure 3:**
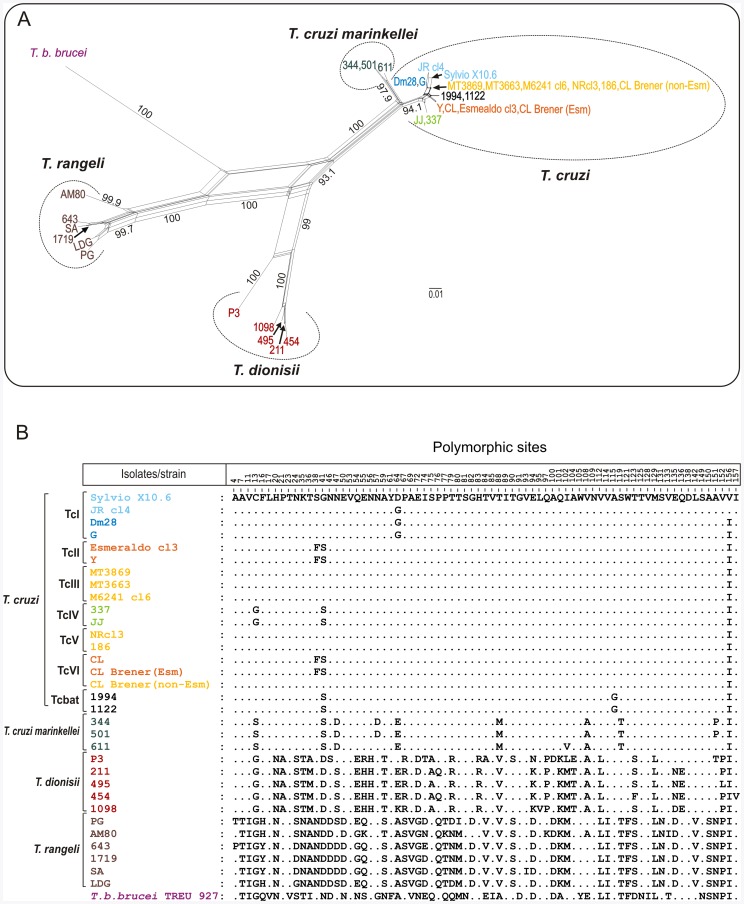
Network and polymorphism analyses on catalytic domain of cruzipain genes from different trypanosome species. Genes from *Schizotrypanum* species (*T. cruzi, T. c. marinkellei* and *T. dionisii*) were compared with homologues from their closest relative species, *T. rangeli,* and the distant related *T. b. brucei*. (A) Network of 33 amino acid predicted sequences constructed using the Neighbour-Net algorithm excluding all conserved sites and with Uncorrected p-distance. The numbers in nodes correspond to bootstrap values from 100 replicates. (B) Polymorphism on cruzipain amino acid sequences from the distinct trypanosome species.

To study the relationships of cruzipain genes from all *T. cruzi* DTUs, *T. c. marinkellei* and *T. dionisii,* and homologues from *T. rangeli* and *T. b. brucei* (Table 1), we aligned ∼500 bp cdCATL sequences obtained in this study with the corresponding genes from *T. cruzi* CL Brener (Esmeraldo-like and non-Esmeraldo-like haplotypes), Esmeraldo cl3, JR cl4, M6241 cl6 and Sylvio X10.6. The analyses of either amino acid (Fig. 3) or nucleotide sequences (data not shown) generated networks of cruzipain genes with similar topologies. Sequences of cd-cruzipain from all *T. cruzi* DTUs always clustered together in a homogeneous assemblage (∼1.2% amino acid sequence divergence) separated from *T. c marinkellei* (5.5% divergence), and largely divergent from the cluster formed by *T. dionisii* from Brazil (20%) and Europe (21%). Amino acid sequences of cruzipain genes of all *Schizotrypanum* species clustered together and largely separated (28%) from the homologous genes of *T. rangeli* and *T. b. brucei* (Fig. 3; Table 1).

**Table 1:** *Trypanosoma cruzi* isolates of all DTUs (TcI-TcVI) and other trypanosome species, and their respective sequences of cruzipain and homologous genes determined in this study or retrieved from data banks.

TCC^a^ code	Trypanosome isolate	Host species		Geographic origin	DTU/genotype	Accession number of cruzipain and homologous sequences	Origin of sequences d
*T. cruzi*							
1321	Dm28	opossum	*D. marsupialis*	Colombia	TcI	JF421288^c^/JF421289^c^	PCR
-	Sylvio X10.6	human	*H. sapiens*	Brazil	TcI	U41454^b^	GenBank
-	JR cl4	human	*H. sapiens*	Venezuela	TcI	TJR4_1_c6805^c^/TJR4_1_c12312^c^	draft genome
30	G	opossum	*D. marsupialis*	Brazil	TcI	JF421290^c^/JF421291^c^/JF421352^b^/JF825059^c^/JF825060^c^	draft genome and PCR
417	M2542	bat	*T. tricolor*	Brazil	TcI	JF421292^c^/JF421293^c^	PCR
507	MO115	bat	*C. perspicillata*	Brazil	TcI	JF421294^c^/JF421295^c^	PCR
34	Y	human	*H. sapiens*	Brazil	TcII	AF314929^b^/JF421310^c^/JF421311^c^	GenBank and PCR
2120	Esmeraldo cl3	human	*H. sapiens*	Brazil	TcII	JF421314^c^/JF421315^c^/scf7180000307932^b^ scf7180000305060^b^/scf7180000304994^b^	TriTrypDB and PCR
844	MT3869	human	*H. sapiens*	Brazil	TcIII	JF421335^c^/JF421336^c^	PCR
845	MT3663	triatomine	*P. geniculatus*	Brazil	TcIII	JF421337^c^/JF421338^c^	PCR
1386	Unidero	dog	*C. familiaris*	Brazil	TcIII	JF421339^c^/JF421340^c^	PCR
-	M6241 cl6	human	*H. sapiens*	Brazil	TcIII	cCM62_C86 b	draft genome*
85	JJ (José Julio)	human	*H. sapiens*	Brazil	TcIV	JF421304^c^/JF421305^c^	PCR
337	Fuscicolis 15	monkey	*S. fuscicolis*	Brazil	TcIV	JF421306^c^/JF421307^c^	PCR
778	Rb778	triatomine	*R. brethesi*	Brazil	TcIV	JF421308^c^/JF421309^c^	PCR
187	Bertha	human	*H. sapiens*	Bolivia	TcV	JF421316^c^ - JF421319^c^	PCR
186	Tc186	triatomine	*T. infestans*	Bolivia	TcV	JF421320^c^ - JF421327^c^	PCR
967	NR cl3	human	*H. sapiens*	Chile	TcV	JF421328^c^ - JF421334^c^	PCR
33	CL	triatomine	*T. infestans*	Brazil	TcVI	JF421312^c^/JF421313^c^/JN701890 c - JN701895 c	PCR
	CL14	triatomine	*T. infestans*	Brazil	TcVI	JF825061^c^ - JF825064^c^	PCR
-	CL Brener Esmeraldo andNon-Esmeraldo haplotypes unassigned contigs	triatomine	*T. infestans*	Brazil	TcVI	Tc00.1047053509429.320^b^/Tc00.1047053507537.20^b^/Tc00.1047053507603.270^b^/Tc00.1047053507603.260^b^/Tc00.1047053507537.10^b^/AAHK01021104^b^/AAHK01015705^b^ AAHK01014707^b^/AAHK01010644^b^ AAHK01012365^b^/AAHK01018585^b^ AAHK01019951^b^	TriTrypDB
294	998	bat	*M. levis*	Brazil	Tcbat	JF421296^c^/JF421297^c^	PCR
499	1336	bat	*M. nigricans*	Brazil	Tcbat	JF421298^c^/JF421299^c^	PCR
1994	MO294	bat	*M. levis*	Brazil	Tcbat	JF421300^c^/JF421301^c^/JF421353^b^	draft genome and PCR
1122	1122	bat	*M. albescens*	Brazil	Tcbat	JF421302^c^/JF421303^c^	PCR
*T. cruzi*	*marinkellei*						
344		bat	*C. perspicillata*	Brazil		JF421354^b^	PCR
501		bat	*C. perspicillata*	Brazil		JF421343^c^	PCR
611		bat	*A. planirostris*	Brazil		JF421344^c^	PCR
*T. dionisii*							
-	P3	bat	*P. pipistrellus*	England		JF421345^c^	PCR
495		bat	*C. perspicillata*	Brazil		JF421346^c^	PCR
1098		bat	*Myotis* sp	Brazil		JF421347^c^	PCR
454		bat	*D. rotundus*	Brazil		JF421348^c^	PCR
211		bat	*E. brasiliensis*	Brazil		JF421355^b^	draft genome
*T. rangeli*					Lineage^e^		
643	Tra643	bat	*P. lineatus*	Brazil	E	FJ997568^c^	GenBank
1719	Tra1719	bat	*A. planirostris*	Brazil	A	JF421351^c^	PCR
031	SA	human	*H. sapiens*	Colombia	A	FJ997556^c^	GenBank
086	AM80	human	*H. sapiens*	Brazil	B	JF421356^b^	draft genome
014	PG	human	*H. sapiens*	Panama	C	FJ997564^c^	GenBank
-	LDG cl1	human	*H. sapiens*	Colombia	C	L38512^b^	GenBank
*T. b. brucei*						-	
-	*T. b. brucei* TREU927	tsetse fly	*Glossina sp*	Kenya		XM_840125^b^	GenBank

a TCC, Code number of the isolates/strains cryopreserved in the Trypanosomatid Culture Collection (TCC); Sequences from cruzipain and homologous genes:

b whole genes,

c catalytic domains;

d cruzipain sequences obtained by sequencing of PCR-amplified genes or from genome databases: GenBank, TriTrypDB and drafts genomes from our Tree of Life project or from the Washington University School of Medicine project*;

e lineages of *T. rangeli* defined by Maia da Silva et al. [22], [61].

### Polymorphism of Cruzipain Gene Copies within Isolates and DTUs of *T. cruzi*

**Figure 4:**
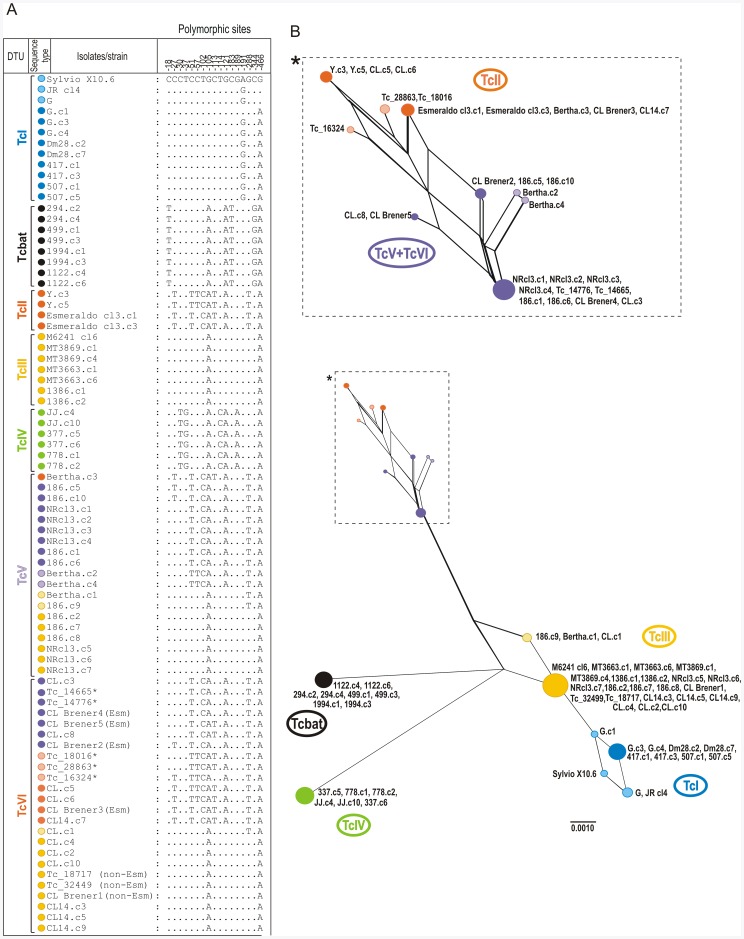
Polymorphism and network analyses of catalytic domain sequences of cruzipain genes from *T. cruzi* isolates of TcI-VI and Tcbat. (A) Polymorphic nucleotide sites on catalytic domains of cruzipain encoding genes; (B) Network based on polymorphic nucleotides constructed with the Neighbour-Net algorithm excluding all conserved sites and with Uncorrected p-distance. The numbers in nodes correspond to bootstrap values from 100 replicates. CLBrener1-5 are sequences from TriTrypDB: Tc00.1047053509429.320, Tc00.1047053507537.20, Tc00.1047053507603.270, Tc00.1047053507603.260 and Tc00.1047053507537.10; *GenBank accession numbers of all sequences included in these analyses are listed on Table1. Major types of sequences from *T. cruzi* isolates of different DTUs are indicated by different colors according to the legend.

Any attempt to associate cruzipain polymorphisms with biological features of *T. cruzi* requires a good appraisal of the diversity of gene copies within both one strain/isolate and each DTU. We have assessed the polymorphism on cruzipain gene copies by comparing 3 to 8 sequences from each isolate. Larger number of sequences (7–8) was analyzed from the isolates of hybrid DTUs TcV and TcVI (Table 1). Cruzipain gene copies (paralogous) from isolates of TcI, TcIII, TcIV and Tcbat were identical or highly similar in their amino acid sequences (Fig. 3), whereas diverged in 2 to 6 polymorphic sites in their nucleotide sequences (Fig. 4). We identified a total of 23 variant sequences of cruzipain sequences (Fig. 4). Relatively homogeneous but not identical copies were found by comparing sequences from 6 isolates of TcI and two of TcII. No polymorphic sites were found among sequences from 3 isolates of each TcIII and TcIV. Sequences from Non-Esmeraldo-like haplotype of CL Brener were identical to those of TcIII (Fig. 4).

Results disclosed high nucleotide polymorphism on cruzipain gene copies of the heterozygous hybrids assigned to TcV (3 isolates, 4 to 8 sequences of each) and TcVI (3 isolates, 24 sequences). Different sequences were found in TcV (at least 6 sequence types) and TcVI (8 types), including sequences identical to those found in TcII or TcIII, sequences found in both TcV and TcVI, and sequences so far detected exclusively in TcV or TcVI (Fig. 3, 4).

### Distinguishing DTUs of *T. cruzi* according to Polymorphisms on Catalytic Domain of Cruzipain Genes

We evaluated the suitability of the polymorphism on nucleotide sequences of catalytic domain (cd) of cruzipain genes in distinguishing among the *T. cruzi* DTUs by comparing 80 sequences from 25 isolates of all DTUs (66 sequences from 22 isolates were determined in this study). Sequences differed by 17 polymorphic nucleotide sites and were divided in 5 major types in addition to the hybrid sequences from TcV and TcVI (Fig. 4). Despite polymorphisms within DTUs, unique polymorphic sites distinguished all DTUs. Cruzipain polymorphisms also distinguished Tcbat from all established DTUs (Fig. 4).

To evaluate the suitability of cruzipain polymorphisms as markers for genotyping, we examined at least three isolates from each DTU, excepting TcVI for which CL Brener and CL 14 were analyzed. Despite multiple copies and small polymorphisms among repeats within the same DTUs and even the same strain (Fig. 4), cruzipain encoding genes showed high sequence conservation of catalytic domains from isolates of the same DTU and clustering of sequences was according to DTUs. Thus, the cruzipain analysis agreed with genotyping methods based on either multiple copy or single copy gene markers [6]. In contrast, large polymorphism and unique sequences detected in each TcV and TcVI (Fig. 4) suggested that these markers can be valuable to detect hybrids. Nevertheless, the use of cruzipain genes for *T. cruzi* genotyping demands the sequencing of several PCR-amplified sequences and comparison of homologous sequences through phylogenetic analyses. A method based on PCR-RFLP analysis of cruzipain genes has been currently developed to facilitate the use of cruzipain as marker for *T. cruzi* genotyping [Lima et al., in preparation].

### Genomic Organization and Synteny of Cathepsin-L Genes in Trypanosome Species

Previous studies showed that cruzipain genes are organized in the genomes of trypanosomes as tandem arrays of duplicated and polymorphic genes located in two or more chromosomes [25]. Analysis of the genomic organization of cruzipain genes in *T. cruzi* CL Brener Esmeraldo-like and non-Esmeraldo-like haplotypes disclosed polymorphisms in number and organization of genes encoding cruzipain (cruzipain, cruzipain 2 and other putative isoforms). There was substantial variation in number, sequence, chromosome and position of the duplicate genes of the two haplotypes; Esmeraldo-like showed 1–4 cruzipain repeats dispersed in three loci and non-Esmeraldo-like present 3–5 copies in three loci (data from TriTrypDB).

**Figure 5:**
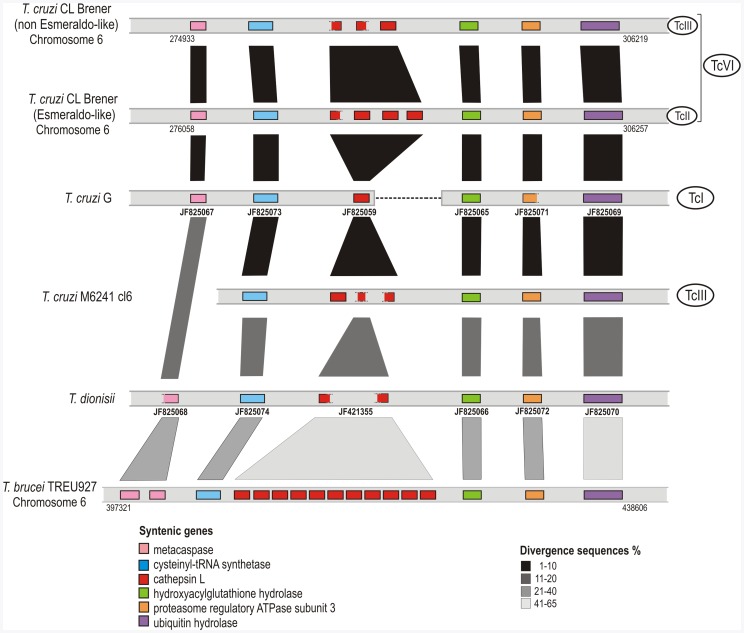
Synteny of a locus containing cruzipain genes in *T. cruzi*, *T. dionisii* and *T. b. brucei*. Segments from the chromosome 6 of *T. cruzi* CL Brener non-Esmeraldo-like and Esmeraldo-like haplotypes, corresponding to TcIII and TcII, respectively, showing 3 to 4 cruzipain gene copies (entire or partial) flanked by orthologous genes marked with different colors according to the legend. Data from the draft assembly of *T. cruzi* G, M6241 cl6 and *T. dionisii* allowed to place one, three or two cruzipain gene copies, respectively, within the same syntenic region (figure do not reflect their actual position on chromosomes). Syntenic region from the chromosome 6 of *T. b. brucei* comprising 11 copies in tandem of brucipain genes was included in the alignment. The shades of vertical gray bars indicate the variable degrees of divergence between sequences according to the legend. The accession codes of all contigs/scaffolds and GenBank accession numbers (in bold) are presented below the corresponding sequences.

Chromosome segments from the genome of *T. cruzi* CL Brener (TcVI) containing three and four tandem copies of cruzipain in Esmeraldo and non-Esmeraldo haplotypes, respectively, were compared with data from the genome drafts of *T. cruzi* G (TcI), M6241 cl6 (TcIII) and *T. dionisii.* Homologous segments of chromosome 6 from *T. cruzi* CL Brener containing cruzipain repeats were found in the genomes of other *T. cruzi* strains: one cruzipain gene copy from *T. cruzi* G (of three copies detected in the genome) and three from M6241 cl6 (6 copies in the genome). In the genomes of these strains, cruzipain and homologous genes were flanked by five orthologous genes thus constituting a syntenic block (Fig. 5). Homologous cruzipain genes arranged in the same order were detected in the genomes of *T. dionisii* (two out of three copies found in the genome draft) and *T. b. brucei* (an array of 11 identical copies of brucipain in the chromosome 1) (Fig. 5). This syntenic block was selected for this study considering the positioning of cruzipain gene copies from distinct *T. cruzi* strains and the synteny shared with *T. dionisii, T. b. brucei* (Fig. 5) and other trypanosome species as showed with *T. vivax* and *T. congolense* genome drafts (data not shown).

Assembly of repetitive sequences in tandem arrays is very problematic, and both the copy number and position are very difficult to be accurately determined. Miss-assembly from collapsed repeat sequence frequently arises during automated genome assembly when small sequence reads originating from distinct repeat copies are incorrectly joined to generate a single unit. This artefact could not be ruled out in the genome drafts analyzed. To avoid mis-assembled genes, we selected contigs containing sequences from both cruzipain and adjacent orthologous genes, which warrant the positioning of the genes encoding cruzipain in this syntenic region (Fig. 5). The degrees of sequence identity between each syntenic gene (Fig. 5) agreed with the phylogenetic relationships among all trypanosome species investigated: *T. cruzi*, *T. c. marinkellei*, *T. dionisii*, *T. rangeli* and *T. b. brucei* [7], [10], [11], [14], [21], [54].

### Comparative Expression Analyses of Cruzipain and Homologues in *T. cruzi*-like and *T. rangeli* by Northern Blot Hybridization and Detection of Proteolytic Activity in Gelatin Gels

**Figure 6:**
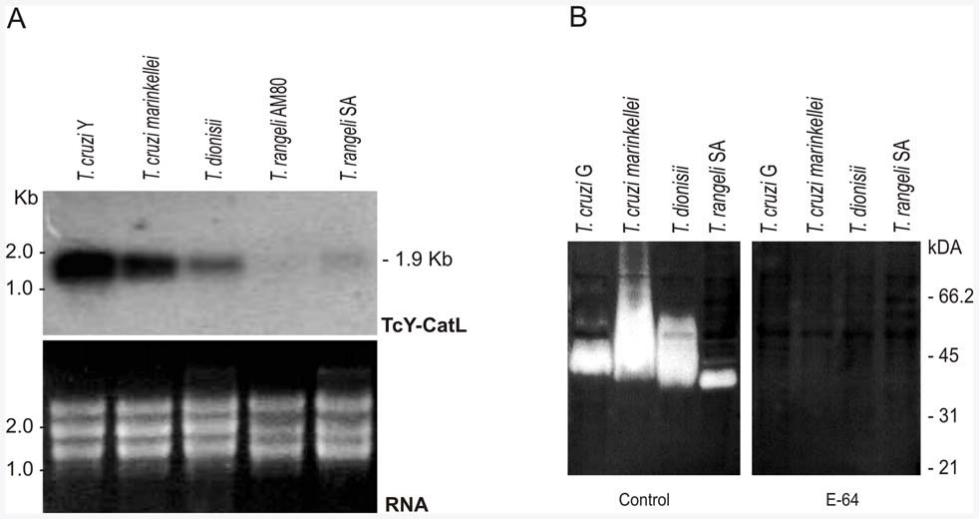
Comparative expression analysis of cruzipain and homologues in *T. cruzi, T. cruzi marinkellei, T. dionisii* and *T. rangeli.* (A) Northern blotting analysis of cruzipain transcripts from *T. cruzi* (Y) and cross-hybridization using the probe consisting of PCR-amplified catalytic domains of *T. cruzi* Y cruzipain labeled with ^32^P T. cruzi Y (TcY-CatL probe); agarose gel of RNA used for this analysis was stained with ethidium bromide (EtBr). (B) CATL proteolytic activities detected in epimastigote lysates of *T. cruzi* G, *T. cruzi marinkellei, T. dionisii* and *T. rangeli*. Activity banding profiles detected in gelatin gels, pH 5.0 and 5 mM DTT were inhibited in gel incubated with 10 µM E-64.

To assess the expression of cruzipain and homologous enzymes from virulent and non-virulent and human-infective or bat-restricted trypanosome species, developing or not within cells, total RNA from epimastigotes of *Schizotrypanum* species (*T. cruzi* Y, *T. c. marinkellei* and *T. dionisii*) and *T. rangeli* was compared by northern-blot hybridization using *T. cruzi* Y derived catalytic domain cruzipain probe that strongly cross-hybridized with RNA from other *T. cruzi* strains (G, CL and JJ) and from all other trypanosome species using low stringent conditions (data not shown). However, hybridization signals using more stringent conditions correlated very well with sequence identity displaying high cross-hybridization signal with transcripts of *T. cruzi*, moderate hybridization with *T. c. marinkellei,* weak hybridization signal with transcripts of *T. dionisii* and lack of hybrization with *T. rangeli* isolates. This analysis allowed estimation of transcripts of similar size (∼1.9 Kb) for all the *Schizotrypanum* species (Fig. 6). In agreement with the differential cross-hybridization among the transcripts of different species, we previously demonstrated that a probe consisting of *T. rangeli* catalytic domain of CATL (rangelipain) strongly hybridized with *T. rangeli* while cross-hybridization with *Schizotrypanum* species was very weak [54].

Cruzipain, a glycoprotein of ∼50–60 kDa, is post-transcriptionally regulated during the *T. cruzi* life cycle [4], [24], [26]. Here, proteolytic activities related to CATL were detected in epimastigote lysates of all *Schizotrypanum* species examined (Fig. 6B). In gels incubated at pH 5.0 with 5 mM DTT (a condition found to be optimal for the detection of cysteine proteases in these trypanosomes), remarkable high activities resolving in the region of ∼55 to 35 kDa were detected in lysates of *T. c. marinkellei* and *T. dionisii.* Lower cruzipain activity was detected for *T. cruzi* G of TcI compared to *T. cruzi*-like trypanosomes (Fig. 6A). We have detected distinct profiles and comparable activity levels for all *T. cruzi* strains such as the virulent Y and CL (TcII) (data not shown) and the non-virulent JJ (TcIV) [54] using the same conditions for the activity assays (data not shown). Weaker activity was detected in *T. rangeli,* confirming lower expression of rangelipain compared to cruzipain as previously demonstrated [54]. All activities were fully inhibited by the cysteine protease-specific inhibitor E-64, thus corroborating the activity of CATL enzymes (Fig. 6B).

## Discussion

In this study, we compared cruzipain-encoding genes of *T. cruzi* isolates representatives of all known subspecific phylogenetic diversity, including all DTUs (TcI-TcVI) and the new *T. cruzi* genotype Tcbat, as well as homologous genes from *T. cruzi*-like species, *T. c. marinkellei* and *T. dionisii,* the closest relatives of *T. cruzi.* Results demonstrated that cruzipain genes from a large set of *T. cruzi* isolates representative of the overall biological and genetic diversity compared with homologous CATL genes from other species in a phylogenetic framework disclosed divergences that closely parallel their phylogenetic diversity [5]–[7], [10], [11], [14], [57]. Our findings revealed species-specific and DTU-specific variability that may be valuable for elucidation of the roles of cruzipain in host-parasite interactions, virulence and pathogenicity evolution.

Isolates of *T. cruzi* show a range of variation in important biological, immunological, pathological (morbidity and mortality) and clinical characteristics [5], [6]. It is likely that much of this variation is probably due to genetic differences among isolates that can be related to specific DTUs. The genomes of the “non virulent” *T. cruzi* Sylvio X10/1 (TcI) and “virulent” *T. cruzi* CL Brener (TcVI) are highly similar in their gene-dense ‘‘core’’ coding regions, which show strongly conserved synteny interspersed with variable repetitive sequences [58]. Large differences within and among DTUs have emerged in several repetitive gene families such as cysteine proteases, mucins, trans-sialidases, surface protease gp63 and amastigote surface glycoproteins (amastins) [58]–[61].

In this study, a relevant conservation of amino acid sequences of genes encoding cruzipain was found among *T. cruzi* of all DTUs, Tcbat and *T. c. marinkellei* compared to more divergent sequences from *T. dionisii.* Cruzipain sequences from all these species tightly clustered together and were more related to rangelipain from *T.* *rangeli* than to brucipain of *T. b. brucei*. Notwithstanding hard efforts to find sequences homologous to cruzipain 2, no sequences were found that displayed the signature residues identifying this isoform [28]. Besides sequences homologous to major cruzipain, which comprised the largest part of sequences detected, our searches in the genome data banks and PCR-amplified sequences disclosed a few heterogeneous sequences closely related to cruzipain 2 [Lima et al., in preparation] but lacking the signature residues reported as characteristics for the archetype of this isoform. Variations in residues in the S2 pocket may account for peculiar activity and substrate specificity of cruzipain isoforms, hence, detailed biochemical studies will be necessary to assess expression, activity and functions in order to verify whether these sequences encode new isoforms or variants of cruzipain 2. In any case, our findings confirmed that phylogenetic analyses are valuable to discover new cruzipain isoforms and/or variants as recently shown for congopain variants through combined genetic and functional approaches [62].

In network genealogies, sequences of cruzipain from the same species, as well as from strains/isolates of the same phylogenetic lineages, always clustered together. Even with the high degree of conservation in cruzipain genes from all DTUs, polymorphisms on nucleotide sequences from the catalytic domain of cruzipain generated 5 branches within *T. cruzi*, each one comprising sequences from one DTU (TcI-TcVI) or Tcbat. In general, sequences from cruzipain gene copies within the same DTU showed small or no variation at all, as we verified for isolates of TcI-TcIV. Data from these DTUs were consistent with their epidemiology and evolutionary histories, with distances among cruzipain genes suggesting that they have had more time segregated from each other than the more recently emerged TcV-TcVI. Isolates of the same DTUs appear to have had a long association with preferential mammalian hosts and vectors and, consequently, naturally circulate separated by biological, ecological and geographical barriers [5]–[7], [63], [64]. TcV and TcVI were confirmed as formed by heterozygous isolates exhibiting polymorphic cruzipain sequences, in addition to sequences identical to those of putative donors TcII or TcIII. In agreement with their hybrid origin, sequences from TcV and TcVI clustered with TcII or TcIII forming a reticulate pattern in the network genealogy of cruzipain genes. Tandem arrays of cruzipain genes in *T. cruzi* CL Brener genome showed sequences apparently derived from TcII (Esmeraldo-like) and TcIII (Non-Esmeraldo-like), even within a single locus. Unique sequences detected in TcV or TcVI strains could be due to the fact that these strains originated from hybridization between strains of TcII and TcIII not included in this study. In addition, more heterogeneous and unique sequences within hybrid strains could emerge post-hybridization through homologous recombination. Our results corroborate that TcV and TcVI resulted from the hybridization between TcII and TcIII as hypothesized before using other markers. Studies have been performed to better understand the role of hybridization events in shaping the genetic diversity within *T. cruzi* [65]–[67]. Our findings confirmed Tcbat as a new *T. cruzi* genotype not yet assigned to any DTU. Sequences from Tcbat clustered closest to TcI but separated from all DTUs as demonstrated with other markers [6], [7], [68].

Genealogy of cruzipain and homologous genes inferred in this study confirmed *T. c. marinkellei* isolates as closest relative and outgroup for *T. cruzi* isolates, as previously shown by examination of other genes including SSUrRNA, gGAPDH and Cytb [10], [14], [22]. In a comparative proteomic analysis, *T. c. marinkellei* was indistinguishable from *T. cruzi* reinforcing their very close relatedness [69]. To date, Tcbat, *T. c. marinkellei* and *T. dionisii* have been found exclusively in bats and all invade and develop in culture cells of a variety of mammals. Tcbat was infective to mice, despite very low virulence. However, these three bat trypanosomes have been reported to be incapable of development in triatomine species commonly infected by *T. cruzi* [7]–[10], [14], [16].

Evolutionary studies of cruzipain and homologous genes from other trypanosome species corroborated phylogenetic relationships based on SSUrRNA and gGAPDH genes of all species investigated: *T. carassii* from fish [70], *T. rangeli* [54], *T. vivax* [71] and *T. theileri* [72], [73], the later three from mammals. Evolutionary relationships of cruzipain and homologues from these trypanosome species indicated that this gene family expanded by successive gene duplications followed by divergences giving rise to genes with greater similarity when originated from the same rather than distinct species/genotypes. This suggests that concerted evolution is a widespread homogenizing force in this trypanosome multigene family. Consequently, CATL-like gene duplicates (paralogous) of all trypanosome species examined clustered by species, i.e. like orthologous genes in different species, as shown for several repeated genes in tandem arrays from African trypanosomes [74]. However, gene conversion and positive selection can generate diversity in sequence, quantity and order among tandemly repeated genes as showed in this and in previous studies on trypanosomatids [59]–[61], [74].

Results from this study disclosed molecular markers able to identify the phylogenetically closely related *T. cruzi* and *T. cruzi*-like species. Reliable identification and knowledge of the diversity and epidemiology of these species are crucial to understand the shared evolutionary history of *T. cruzi* and *T. cruzi*-like bat trypanosomes [7], [10], [11], [14], [57]. We have previously shown that *T. cruzi* and *T. rangeli* [54], *T. vivax* [71] and *T. theileri* [72] could be diagnosed by specific PCR assays targeting cruzipain sequences. In this study, we demonstrated that cruzipain sequences clustered *T. cruzi* isolates according to DTUs, similarly to CATL-like gene based genotyping of lineages within *T. rangeli* [54], *T. vivax* [71] and *T. theileri* [72], [73]. Altogether, these previous studies and results herein described support the use of these genes as valuable markers for both inter- and intra-species phylogenetic analyses of trypanosomes.

There are clear evidence that immunization with cruzipain catalytic domain, and not with C-terminal domain, confer important cellular protective immunity against *T. cruzi,* as evidenced by the reduction in parasitemia, tissue parasitism and mortality in mice, indicating the existence of epitopes important for protective immunity in the catalytic domains and, thus, supporting the use of cruzipain for vaccine development [34]–[37]. The main goal of all vaccination strategies should be a polyvalent vaccine against infection by different strains of *T. cruzi* of any DTU. Although the polymorphism among cruzipain catalytic domains expressed by distinct strains appears to be reduced, for vaccination purposes it is important to evaluate strain-variant and conserved cross-reactive epitopes in different *T. cruzi* DTUs, and among strains of the same DTU, and the possible relevance of epitope polymorphism for species- or strain-specific protective immune response [75]–[77]. Therefore, knowledge on the genetic diversity within the plethora of DTUs and strains of any antigen vaccine candidate for Chagas disease can be decisive for the design of efficient and polyvalent vaccines using, if necessary, a pool of stage- and strain-specific antigens. Here, we showed for the first time that all the six DTUs, and even different strains of DTUs, exhibited specific genetic variants of cruzipain. Any possible implications of the cruzipain polymorphisms to warrant efficient cross-protection of a potential vaccine require investigations to demonstrate the immunogenicity of cruzipain variants from different *T. cruzi* DTUs.

Drugs currently employed for the treatment of Chagas disease have serious limitations due to their failure in chronic patients and side effects. Inhibitors of cruzipain kill the parasite and cure infected mice, thus validating this enzyme as a very promising target for design of new drugs [1], [2], [4], [43], [44]. In this study, we demonstrated that substrate-binding subsites of cruzipain are conserved in all *T. cruzi* DTUs, but diverge in *T. dionisii* and other trypanosome species. *T. c. marinkellei* cruzipain share identical subsites and, hence, can be a valuable non-infective to human model to test drugs against trypomastigotes and intracellular amastigotes. The efficacy of the cruzipain inhibitor K777 was evidenced against 6 *T. cruzi* strains differing in tissue tropisms and drug susceptibility [44]. More strains must be tested to verify the efficacy of inhibitors for the ample diversity of cruzipain variants expressed by *T. cruzi* of the different DTUs.

Characterization of genes encoding major cruzipain, genealogies of genetic variants and genomic organization were addressed in this study for the first time to compare *T. cruzi* of all DTUs and closest related *T. cruzi*-like species. This is the most comprehensive study using sequences from protein-encoding genes to compare isolates of *T. cruzi* from all DTUs. Of the strains selected for genome sequencing CL Brener and Esmeraldo are virulent whereas Sylvio X10.6 and G strain are considered of low virulence. Most *T. cruzi* isolates examined here by PCR-amplification of cruzipain genes were also previously analysed regarding virulence for mice: TcI, Tcbat and TcIV isolates were non-virulent whereas TcIII (TCC1386) and TcV (TCC197) isolates showed to be virulent for mice (data not shown). Interestingly, non-virulent CL14 clone was shown in this study to be a hybrid strain like the virulent CL Brener [52]. All the six DTUs were supported by cruzipain gene markers. Infections caused by different strains of *T. cruzi* from distinct DTUs extensively diverged in the morbidity and mortality. Attempts to associate *T. cruzi* DTUs with behavioral phenotypes, including virulence and pathogenicity in mice, metacyclogenesis and cell infectivity, suggested an important degree of association [5], [6], [60]. However, besides some overlap between different DTUs, there is a broad intra-DTU phenotypic diversity (largest differences have been reported within TcI) and even among clones of a given strain such as the virulent CL Brener and non-virulent CL14 clones, both of TcVI. Therefore, we can associate cruzipain polymorphisms to DTUs. However, available data are insufficient to support strong correlations between behavioural phenotypes and DTUs. This goal will require extensive studies of *T. cruzi* strains from all DTUs through a combination of phylogenetic, biochemical, pathological and immunological approaches.

Our findings indicated conserved major cruzipain in *T. cruzi* of all DTUs while other trypanosome species express diverse homologous enzymes. Results showed, for the first time, the expression of cruzipain transcripts by *T. c. marinkellei* and *T. dionisii* that correlated very well to sequence divergences among all trypanosome species investigated and, in addition, revealed high proteolytic activity in these two non-pathogenic species. An understanding of the cruzipain gene repertoires, expression and functions can help to elucidate the evolutionary history that shaped variability within *T. cruzi* and its divergence from *T. cruzi*-like species and the more distantly related *T. rangeli*. *T. cruzi*-like trypanosomes, despite sharing genomic and proteomic features [10], [11], [14], [69], exhibit many peculiarities that can potentially explain differences in host preference/restriction, virulence and pathogenesis. Results from this comprehensive study on major cruzipain isoform are the initial steps toward understanding the roles played by genetic repertoires of cruzipain enzymes and homologues in the life cycles and infections caused by *T. cruzi* of all DTUs, *T. cruzi*-like species and *T. rangeli*.

## Materials and Methods

### PCR Amplification, Sequencing and Phylogenetic Analysis of CATL Sequences

The whole sequences of cruzipain genes (∼1.4 Kb) of *T. cruzi* Esmeraldo cl3 and CL Brener were retrieved from TriTrypDB. Homologous genes in *T. cruzi* M6241 cl6 (Project ID:59941) were obtained from the genome drafts produced by the Genome Institute at Washington University School of Medicine (St. Louis, USA). *T. cruzi* G, Tcbat 1994, *T. dionisii* 211 and *T. rangeli* AM80 were obtained from genome drafts that we are currently performing using standard pyrosequencing shotgun methodology according to Roche 454 protocols as described previously [78]. Resulting reads were submitted to Roche’s Newbler software (version 2.3) and contigs containing genes described in this study were assembled in scaffolds (Table 1). Sequences retrieved from genome data banks were aligned with homologues from *T. cruzi* Sylvio X10.6 and Y, *T. rangeli* LDG and *T. b. brucei* TREU 927 from GenBank and *T. c. marinkellei* 344 determined in this study. The whole sequence from *T. c. marinkellei* was obtained by PCR-amplification using the primers TDIO5-FOR (5′ ATG ACG AGC TGG GCG CGT G 3′) and CATL3REV2 (5′ TTA GCT TCA GGA GCG GCG ATG 3′) and the conditions described previously [54], [71]. Cathepsin L sequences determined in this study are available in GenBank (Table 1).

PCR products from catalytic domain of cruzipain genes (cd-cruzipain) obtained using primers and reaction conditions described previously [54], [71] were cloned, and 3 to 8 clones from each species/isolates were sequenced. Alignment of sequences encoding cd-cruzipain genes includes 14 sequences from data banks and 66 determined in this study of all DTUs (TcI-TcVI), Tcbat and homologues from *T. c. marinkellei* and *T. dionisii* from Brazil and England [7], [14]. Sequences from non-*Schizotrypanum* trypanosomes, *T. rangeli* isolates (SA, AM80, PG, 643, 1719 and LDG) representative of its major lineages [21], [22], and *T. b. brucei* were also added in the alignment. Sequences determined in this study reflecting the spectrum of genetic polymorphism observed were deposited in GenBank (Table 1). All sequences employed in this study are listed in Table 1.

We initially performed a broad search in all available genomes aiming to detect cruzipain isoforms. Most sequences were homologous to the major cruzipain, and no sequences showed the amino acid residues typical of cruzipain 2. A significant number of sequences from distinct strains of *T. cruzi,* including those obtained by PCR-amplification were found to be different from cruzipain and cruzipain 2 (data not shown). For genealogy analysis, we selected only sequences with the signatures of cruzipain (Fig. 1), thus removing some sequences of other putative isoforms. Sequences were aligned using Clustal X [79] and manually adjusted. The amino acid sequence of cruzipain was used as a template to ensure codon-to-codon correspondence. Phylogenetic relationships were inferred using nucleotide and predicted amino acid sequences from the entire genes or restricted to pre-pro or cdCATL domains. Network genealogy was inferred in SplitsTree v4.11.3 using the neighbor-net method [80]. Internode supports were estimated by performing 100 bootstrap replicates using the same parameters optimized for network inferences.

### Synteny and Codon Analyses

Syntenic genes flanking CATL genes were identified in the genomes of *T. cruzi* CL Brener (Non-Esmeraldo-like and Esmeraldo-like haplotypes) and *T. b. brucei* 927 genomes (TriTrypDB). Scaffolds comprising cruzipain genes from *T. cruzi* G and M6241 cl6 and homologues from *T. dionisii* were aligned with these syntenic segments. Search on the draft genome of Sylvio X10/1 [58], Tcbat 1994 and *T. rangeli* AM80 disclosed cruzipain sequences, most partial, in small reads that could not be positioned in homologous scaffolds. The ratio of non-synonymous to synonymous (*dN/dS*) amino acid changes was calculated according to Yang and Nielsen [81] using PAML, v.4.2 software to infer relative selection pressures [82].

### Northern Blot and Proteolytic Activities of Cruzipain

For Northern blot analysis, 10 pg of total RNA extracted using Trizol (Gibco BRL) from epimastigotes of *T. cruzi* Y, *T. c. marinkellei* 344, *T. dionisii* 211 and *T. rangeli* isolates AM80 and SA was electrophoresed in 1.0% agarose gel and blotted onto nylon membrane. The membrane was hybridized for 14–16 hr at 40°C with a probe consisting of PCR-amplified cd-cruzipain of *T. cruzi* Y labeled with ^32^P and washed in 1×bufffer (0.3 M NaCl, 0.3 mM Na citrate, pH 7.0 containing 0.1% SDS) at 50°C for 1 h as described previously [54].

To assess the proteolytic activities in gelatin gels, lysates of cultured epimastigotes were subjected to electrophoresis in 10% resolving SDS-acrylamide gels containing 500 µg/ml gelatin as described before [54], [72]. Briefly, gels were incubated in 2.5% (v/v) Triton X-100 in 0.1 M buffer (acetate: pH 4.0 and 5.0; Tris-HCl: pH 7.5) containing 5 mM DTT (dithiothreitol) and then, for ∼18 h in buffer-DTT. Maximum activity was detected in pH 5.0 and was greatly stimulated by DTT. Bands associated to cysteine proteases were identified by incubating gel halves with 10 µM E-64. Gels were fixed with 10% TCA, and stained with Coomassie blue R-250 [54], [72].
